# Conserved residues in the Gα interface show subtype specificity in Gβγ coupling

**DOI:** 10.1016/j.jbc.2026.113231

**Published:** 2026-06-04

**Authors:** Wenyuan Wei, H. Dalton Taylor, Ning Ma, Andrei S. Rodin, Sergio Branciamore, Henrik G. Dohlman, Nagarajan Vaidehi

**Affiliations:** 1 Department of Computational & Quantitative Medicine, Beckman Research Institute of the City of Hope, Duarte, California, USA; 2 Irell and Manella Graduate School of Biological Science, Beckman Research Institute of the City of Hope, Duarte, California, USA; 3 Department of Pharmacology, University of North Carolina at Chapel Hill, Chapel Hill, North Carolina, USA

**Keywords:** molecular dynamics simulations, bioluminescent resonance energy transfer, Bayesian Network Model, heterotrimeric G proteins, site-directed mutagenesis

## Abstract

Although the structural basis of selective interaction of G-proteins and G protein-coupled receptors (GPCRs) is well-characterized, the mechanisms underlying selective interactions between distinct Gα subtypes (Gα_s_, Gα_i_, Gα_q_, Gα_12_, *and so on*) and Gβγ remain poorly understood. While conserved residues in Gα subtypes are often assumed to have similar functions, they may instead modulate coupling selectivity by altering the frequency and stability of contacts at the Gα:Gβγ interface. Using molecular dynamics (MD) simulations combined with the interpretable machine learning method, Bayesian Network Model (BNM), and protein–protein proximity (BRET) assays, we show that conserved residues in the two closely related Gα_i/o_ and Gα_q/11_ subfamilies contribute differentially to Gβγ coupling. These conserved residue “hotspots” on Gα_i1_ and Gα_q_ produced divergent functional effects on Gβγ coupling, indicating that conservation does not ensure functional equivalence. These findings suggest that local microenvironment and paralog-specific allosteric coupling shape how conserved interface residues contribute to protein-protein coupling. The framework provides a systematic approach for dissecting subtype-specific mechanisms, with implications for drug design and for annotating the functional relevance of disease-associated variants. The computational methods used here are broadly applicable to other homologous protein families.

Analyses of protein paralogs provide a direct test of whether conserved residues play a similar role in functionally similar proteins. This is a central tenet of the sequence–structure–function paradigm. Although the assumption that conserved residues contribute similarly to protein-protein interactions holds in some cases, reported exceptions indicate that conservation alone does not guarantee functional equivalence (1, 2). These observations suggest that local microenvironments and long-range allosteric communication can modulate how individual residue positions contribute to signaling outputs. Previous studies have shown that conserved residues in the GPCR:Gα interface can indeed contribute differentially to Gα subtype coupling by GPCRs (3). Here we consider conserved residues that contribute to the coupling of Gα subtypes to Gβγ. We use the term “coupling” interchangeably with “interaction”, defined as the binding between two protein partners.

The Gα subunit of the trimeric G protein is a GTPase that dissociates from Gβγ upon activation and engages downstream pathways. Humans encode 16 Gα subtypes, functionally categorized into four subfamilies (Gα_s_, Gα_i/o_, Gα_q/11_, Gα_12/13_), and these couple with differential strengths to receptors. However, it is not known whether conserved residues in Gα subtypes contribute to coupling with differential strength to Gβγ. Structurally, Gα proteins consist of two domains: a Ras-like domain with GTPase activity and an α-helical domain (AHD) that participates in nucleotide binding. Within the Ras-like domain, three switch regions (SWI–III) rearrange upon GTP binding, promoting Gα-Gβγ dissociation (4). The nucleotide-binding pocket lies between the Ras domain and the AHD, which undergoes opening upon GPCR-mediated activation of Gα. Despite broad conservation in sequence and structure, Gα subfamilies trigger distinct effector pathways: Gα_s_ activates adenylyl cyclase to elevate cAMP (5); Gα_q/11_ stimulates phospholipase Cβ isoforms to produce intracellular Ca^2+^ signals (6); Gα_12/13_ activates RhoGEFs to regulate Rho-family GTPases (7); and Gα_i/o_ inhibits adenylyl cyclase and activates GIRK channels (5, 8).

Due to the larger number of distinct GPCRs in comparison to the number of G protein subtypes and downstream effectors, G proteins represent a critical bottleneck in GPCR-mediated signaling pathways. Perturbations of G protein function, especially that of Gα, can therefore impact multiple signaling pathways. Indeed, many reported Gα mutations are deemed disease-associated. For example, Gα_s_ substitutions at Arg201 (*e.g.*, R201C), which slow GTPase activity, are linked to McCune–Albright syndrome and various cancers (9, 10, 11). More than 200 unique variants of Gα_o_ are linked to epileptic encephalopathy; these mutations are scattered across the AHD and Ras-like domain and fall into multiple mechanistically distinct groups (12). In our previous study (13) we showed that one of these mutations, in a conserved arginine in an evolutionarily conserved Gly-Arg-Glu triad (“G-R-E motif”), impede Gβγ subunit dissociation. We showed that this triad forms an allosteric link between the γ-PO_4_ group of GTP and the Gβγ interface on Gα. Substitutions at a conserved “catalytic glutamine” essential for GTP hydrolysis, in Gα_q_ or Gα_11_ are found in nearly all cases of uveal melanoma (14, 15). Hewitt-Valentin *et al.* showed that different substitutions at the catalytic Gln produce distinct active-state conformations of Gα (1). That study showed that Gα function arises from an ensemble of active states, some of which are preferentially selected in disease and therefore may be differentially targeted by receptor-directed ligands. Furthermore, substitutions at this conserved Gln in Gα_q_ confer an allosteric impact different from the equivalent substitution in Gα_i1_, indicating distinct allosteric communication mechanisms across different Gα subtypes.

Here, we examined whether conserved residues in the Gα:Gβγ interface, in two closely related subfamilies, Gα_i/o_ and Gα_q/11_, have differential effects on Gβγ coupling. We chose Gα_i1_ and Gα_q_ since they are representative of the two most evolutionarily related Gα subfamilies (16), and exhibit high sequence, size and structural similarity (Figs. 1 and S1). The differences in their contribution to Gα:Gβγ interface coupling strength could come from spatiotemporal (spatial and time-dependent) dynamic elements of the residue contacts in the interface. Therefore, we used a dynamic conformational ensemble generated from all-atom Molecular Dynamics (MD) simulations of the trimeric Gα_i1_ and Gα_q_ and analyzed the properties of the persistence of residue contacts, enthalpy of the interaction strength, and cooperativity using Bayesian Network Model (BNM), an interpretable unsupervised machine learning model. We predicted the existence of conserved residues that contribute differentially to Gα_i__1_ and Gα_q_ coupling to Gβγ and tested the predictions using bioluminescence resonance energy transfer (BRET) assays for Gβγ dissociation from the activated Gα subunit. Our results indicate that conserved positions can support subfamily-specific allosteric effects at the interface. These conserved subtype-specific hotspot residues may represent key nodes in the allosteric communication network that distinguishes Gα_i1_ from Gα_q_.

**Figure 1:**
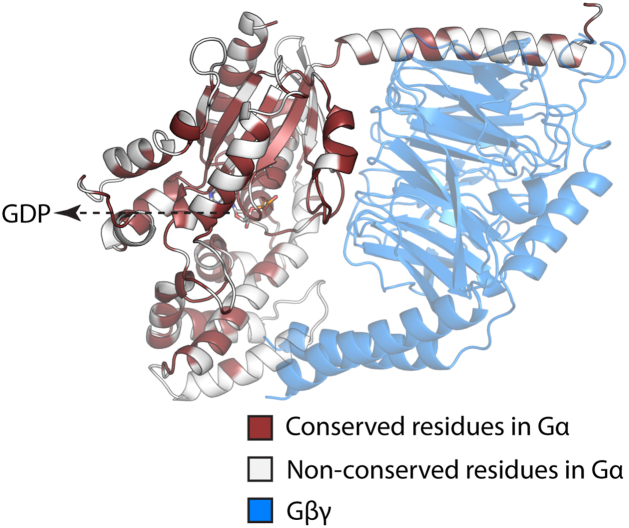
**Illustration of conserved residues between Gα_i1_ and Gα_q_.** GDP is shown in *sticks*. The residues conserved between Gα_q_ and Gα_i1_ are shown in *maroon* and the non-conserved residues in *grey*. The Gβγ subunits are shown in *blue*.

## Results

### Contact frequencies and interaction energies of the interface residues show subtype-specific hotspots in the α2 helix of Gα_i1_ and Gα_q_

In this study, we investigated the role of conserved residues in the interface of Gα_i1_ and Gα_q_ with Gβγ. Our goal is to identify the conserved residues in the Gα interface with Gβγ that show subtype-specific behavior. We define a hotspot as a residue that, upon mutation, affects the coupling of Gα to Gβγ significantly. Furthermore, a common hotspot is defined as any residue that affects Gβγ coupling in both Gα_i_ and Gα_q_ subtypes equally, whereas a subtype-specific hotspot affects one Gα subtype more than the other.

**Figure 2:**
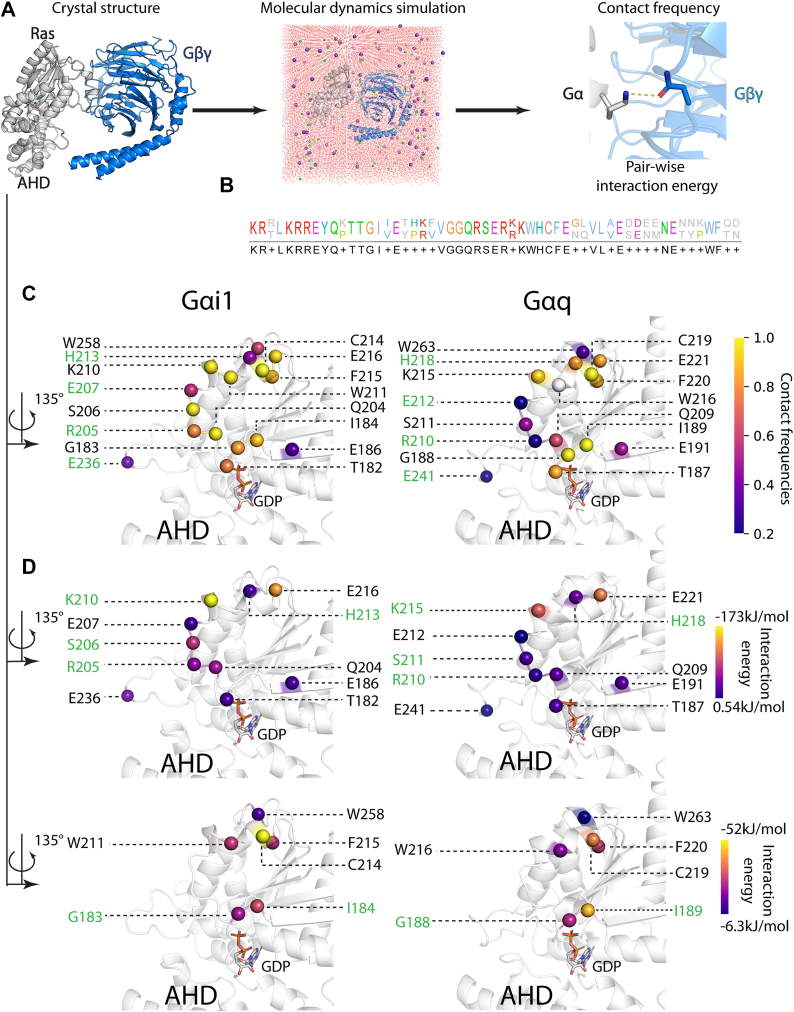
**Thermodynamic properties of the interface residues in Gα_i1_ and Gα_q_ with Gβγ are highly similar.***A*, workflow showing how the MD simulations were constructed from the starting structure, and how contact frequencies and interaction energies were obtained from the trajectories. *B*, sequence alignment of Gα interface residues. Top row is Gα_i1_, and bottom row is Gα_q_. The residues are color-coded by their physiochemical properties. *Blue*: hydrophobic, *red*: positively charged, *purple*: negatively charged, *green*: polar uncharged, *yellow*: cysteine, *orange*: glycine. *C*, structural location of the residue contacts colored by contact frequencies of the conserved residues on the Gα subtypes. *Green labels* indicate residue contacts with statistical significance between the Gα_i1_ and Gα_q_ across 5 independent MD trajectories (two-tailed Welch’s *t* test, *p* < 0.05; see Experimental procedures). *D*, Location of the residue contacts colored by their interaction energies on the interface. *Top*: polar residues, *bottom*: non-polar residues. *Green labels* indicate statistical significance between Gα_i1_ and Gα_q_ across 5 independent MD trajectories (two-tailed Welch’s *t* test, *p* < 0.05).

We performed MD simulations on the trimeric G protein complexes starting from their respective crystal structures (Gα_i1_: 6CRK; Gα_q_: 7F6G; Fig. 2*A*). We performed five independent MD simulations each 1 μs long, totaling 5 μs for both Gα_i1_ and Gα_q_ and tested each run for convergence (see Experimental procedures section). For each MD run, we calculated the contact frequencies of residue pairs in the Gα:Gβγ interface. The contact frequency is the percentage of MD snapshots that show a given residue contact in the interface. We also calculated the pair-wise interaction energies of the residue contacts in the Gα:Gβγ interface (Fig. 2*A*). Subsequently, we derived the per-residue contact frequencies and interaction energies for Gα residues from the above pairwise interactions (see Experimental procedures).

There are 50 amino acids in Gα_i1_ that contact Gβγ in the interface, and 59% of these residues in Gα_i1_ are strictly conserved with Gα_q_ (Fig. S2*A*). There are 47 residues in Gα_q_ that contact Gβγ in the interface, and 66% of these residues in Gα_q_ are conserved with Gα_i1_ (Fig. S2*A*). The polar contacts are modestly enriched in Gα_i1_ relative to Gα_q_, whereas the number of non-polar residues contacting Gβγ is comparable between the subtypes (Fig. S2*B*).

We analyzed conserved interface residues that have contact frequencies of more than 20% (hereafter referred to as persistent contacts; Fig. S2*C*). Sixteen residues are conserved and made persistent contacts, out of which six are non-polar, and ten are polar. Unpaired *t* test showed that 4/16 residues (25%) that show statistically significant differences in contact frequencies are polar residues (green-labeled residues in Fig. S2*C*). Structural mapping showed that these residues cluster predominantly within the Ras-like domain, consistent with its established importance in Gα:Gβγ interactions (4) (Fig. 2*C*). Residues that show differences among subtypes are located on the α2 helix and the β4-α3 loop; these two regions are toward the periphery or outer rim of the Gα:Gβγ interface. This finding is reminiscent of our previous study showing that residues conferring Gα coupling specificity to GPCRs are likewise located in the outer rim of the Gα:GPCR interface (17).

A larger set of conserved residue contacts in the Gα:Gβγ interface showed subtype-specific differences by interaction energy analysis. Because polar and nonpolar residues have distinct magnitudes of interaction energies, we analyzed them separately (Figs. 2*D* and S2*D*). 40% of the polar (Fig. S2*D*) and 33.3% of the non-polar interface residues (Fig. S2*C*) showed a statistically significant difference in interaction energies. We observed two common residues (R205/R210, H213/H218) that had statistically significant differences in contact frequencies and interaction energies. Overall, measures of interaction energies revealed more differences than those of contact frequencies.

All residues that showed differences in either contact frequencies or interaction energies are also highly conserved across species. There is clinical evidence that residue positions G183/G188, I184/I189, S206/S211, and E236/E241 are linked to the development of encephalopathies when mutated in Gα_o_, a subtype closely related to Gα_i1_ (12, 18). These data show that contact frequency and interaction energy, when taken together, reveal subtype-specific hotspots, some of which are already reported to be mutational hotspots.

### Bayesian Network Analysis reveals local and allosteric co-dependencies in dynamics on Gα:Gβγ interface in Gα_i1_ and Gα_q_

We have previously shown that co-dependence in the dynamic movement or correlated movement among the residues in the interface are important in protein-protein coupling and specificity (19, 20). Residue pairs that show a high level of co-dependence in their movement with multiple residues in the interface are said to be cooperative. We next sought to identify the residue contacts in the Gα:Gβγ interface that showed cooperativity in their dynamic movement. To this end, we applied our previously developed interpretable and unsupervised BNM-based machine learning model, BaNDyt, to analyze MD simulation trajectories (19). BN model analyzes the large-scale data presented in the MD simulation trajectories to generate a graphical network in probabilistic space (20) where the nodes in the network are residues or residue contacts, and the edges connecting the nodes are the probability of co-dependent motions between the nodes. Previously, we have used BN models to predict and test the amino acid residue contacts that show high cooperativity and their allosteric effect in the interface of GPCR:G protein complexes (20). We have developed highly scalable and computationally efficient software packages for generating BN models from MD simulation trajectories: BaNDyT (specialized BN software for the MD data, freely available for download from GitHub) (19, 21, 22, 23).

**Figure 3:**
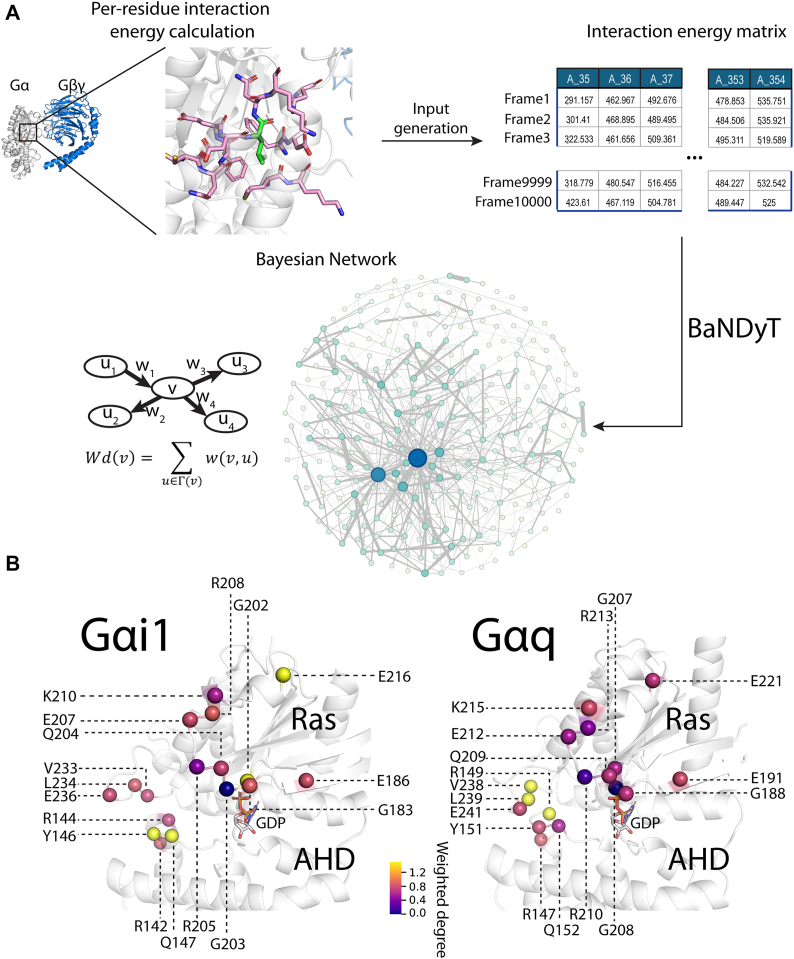
**BNM shows co-dependencies in the dynamic movement among residues in the Gα:Gβγ interface and between different structural regions of Gα subunits of Gα_i1_ and Gα_q_.***A*, schematic of the workflow used for generating the BNMs for Gα_i1_ and Gα_q_ trimers. *B*, Gα-Gβγ interface residues that are also top residues projected on the three-dimensional structure. Colors of the spheres indicate the value of their weighted degrees.

The workflow we used to generate the BN models is shown in Figure 3*A* (see Experimental procedures for details). Briefly, the interaction energy of each residue in the Gα subunit within 12 Å of all other residues in the G protein trimer, including Gβγ residues, and nucleotide was calculated per MD frame, discretized using Max Entropy algorithm into eight bins (15). The resulting matrix was fed to BaNDyT software to generate the BN model. In the resulting network, the sum of all edge weights of a node (residue) is referred to as weighted degree (WD). Gα residues were ranked by their WD, and the residues in the top quartile of WD (hereafter referred to as “top residues”) were selected for further analysis (Fig. S3*A*). We analyzed the conserved residues in the Gα:Gβγ interface that are also in the top quartile by WD. While the number of residues in the top quartile are similar in Gα_i1_ and Gα_q_, they are not the same residues. The three topmost residues (R144, V233, and L234) are unique to Gα_i1_, while four other high-scoring residues (Y151, Q152, G207, and E221) are unique to Gα_q_ (Fig. S3*B*). These residues are highly conserved among Gα_i1_ and Gα_q_ orthologs (Table S1). Unlike subtype-specific hotspots found in contact frequencies and interaction energies, most residues have not been reported to have functional importance in Gα_i1_ or Gα_q_. The exception is G202/G207, which was reported to be important for Gβγ subunit dissociation in Gα (12, 24).When projected to the three-dimensional structures, we observed clustering of high WD residues in the Ras-AHD interface (αD-αE loop and β4-α3 loop; Fig. 3*B*); these two regions separate during domain opening movements, which may confer the high co-dependencies observed.

Using BaNDyT, we identified 7 Gα:Gβγ interface residues that showed differences in cooperativity when comparing Gα_i1_ and Gα_q_ but did not show differences in the contact frequencies and interaction energies (R144/R149, Y146/Y151, Q147/Q152, G202/G207, E216/E221, V233/V238, L234/L239, Fig. S3*C*). The lack of overlap between the subtype-specific hotspots identified using BaNDyT and those identified in residue contact frequencies and interaction energies suggests that an interplay of all three factors could be important for determining the subtype specificity. Of course, other factors such as the complex cell environment, are also important and not considered in this study. We deduced that an integration of the three features could give us a more comprehensive understanding of the intrinsic structural and dynamical features regulating the Gα:Gβγ interface.

### Distance-based prediction of residues as subtype-specific hotspots

**Figure 4:**
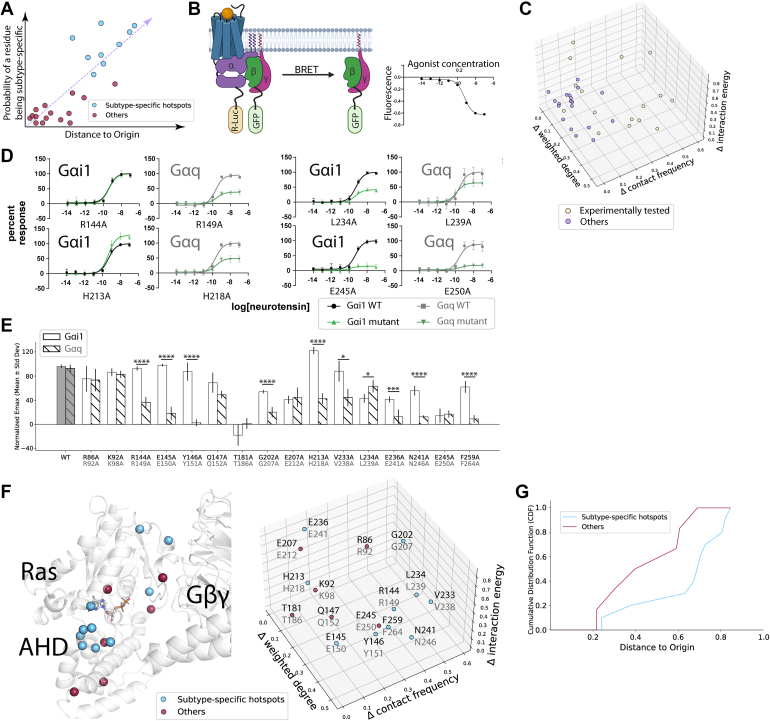
**Distance-based prediction of subtype-specific hotspots and validation *via* TRUPATH BRET assay.***A*, illustration of the hypothesis that the further from the origin, the more likely a residue is a subtype-specific hotspot. All points shown are conceptual and are not actual data. *B*, schematics of the TRUPATH BRET assay. A representative conceptual readout (not actual data) is shown on the right side. *C*, three-dimensional location of each residue with respect to the origin. *D*, TRUPATH BRET dose-response curves of representative mutants. Mean values (±S.E.M.) of three independent experiments were plotted for each agonist concentration, with two replicates per experiment, for a total sample size of 6. *Black*/*gray* curve is the wildtype, and the *green*/*forest* curve is mutant. *E*, normalized E_max_ values (mean ± S.D.) of wildtype and mutants. Stars represent statistical significance using two-tailed Welch’s *t* test (∗<0.5, ∗∗∗<0.005, ∗∗∗∗<0.001). *F*, Projection of residues on the three-dimensional protein structure (*top*), and annotation of tested residue in the Cartesian coordinate as seen in panel (*C*). *Cyan* represents subtype-specific hotspots. *Red* represents other conserved residues in the interface that do not show a subtype-specific effect in the two subtypes. *G*, cumulative distribution function (CDF) curve of residues experimentally validated as subtype-specific hotspots and those that were not (Others), as the distance to origin increases. CDFs of each group represent the accumulated proportion of values up to each distance to origin threshold, illustrating the distributional shifts between the two groups.

Since no single feature was sufficient to distinguish the hotspot residues differentiating Gα_i1_ and Gα_q_, we inferred that subtype-specificity likely arises from a combination of the three residue-based features. Since the raw values of the three properties calculated here are of different units/dimensions, we ranked the interface residues in the quantile analysis of each property to place the features on a comparable distribution. We then calculated the relative ranking of residues within each subtype (Fig. S4*A*). If a residue ranked the same in the two subtypes, it would have zero values for all three features and reside at the origin (Δ = 0,0,0). We hypothesized that the further from the origin, the more subtype-specific a residue is in the calculated features, and the more likely that said residue will show subtype-specific effects between Gα_i1_ and Gα_q_ (Fig. 4*A*).

To test our hypothesis, we selected 16 conserved residues along the Gα–Gβγ interface that are far from the origin, and we mutated each to alanine (Fig. 4*C*). We then used BRET to test the functional importance of these residues. Briefly, Gα and Gγ were tagged with a luciferase donor and GFP acceptor, respectively. In the inactive trimeric state, the BRET ratio, acceptor signal divided by donor signal, is high; upon ligand-induced activation of the receptor, Gα and Gβγ dissociate, and the BRET ratio decreases (Fig. 4*B*). To minimize the number of variables, we compared Gα_i1_ and Gα_q_ subtypes (and their mutants) using the same receptor and agonist. We selected neurotensin receptor type 1 (NTSR1) specifically because it exhibits extreme promiscuity, activating at least twelve G protein subtypes across 3 families. The only G protein family that NTSR1 does not activate well in this BRET platform is Gα_s_ (25, 26, 27). When we compared the dose-response curves (Figs. 4*D* and S5), three distinct patterns in BRET intensity emerged: (i) subtype-specific hotspots that only affected Gα_q_ (*e.g.*, R144A/R149A), (ii) subtype-specific hotspots that affected both subtypes, but to a different extent (*e.g.*, L234A/L239A, H213A/H218A), and (iii) common hotspots that affected both subtypes equally (*e.g.*, E245A/E250A).

We observed that more mutations affected the function of Gα_q_ than Gα_i1_ (Fig. 4*E*). Out of the sixteen residues tested, nine were hotspots within Gα_i1_ (Fig. S4*B*), and fourteen were hotspots within Gα_q_ (Fig. S4*C*). Among the sixteen residues, ten were subtype-specific hotspots: Gα_i1_/Gα_q_ residues R144/R149, E145/E150, Y146/Y151, V233/V238 affected only Gα_q_, and G202/G207, H213/H218, L234/L239, E236/E241, N241/N246, F259/F264 affected both subtypes to different extents. Spatially, they are in three different structural regions of the Gα protein. G202/G207 and H213/H218 are in the interface between the Ras-like domain and Gβγ, whereas F259/F264 is located closer to the Gα:GPCR interface. Most importantly, the rest of the subtype-specific residues are clustered on the interface between the Ras-like domain and the AHD (Fig. 4*F*). This “clamp” region not only contacts the N-termini of Gβ and Gγ, but also undergoes conformational changes in the nucleotide exchange step, leading to opening of the Ras-like domain and AHD (28, 29).

To test our hypothesis that subtype-specific hotspots are located further from the origin (Δ = 0,0,0) than common hotspots, we generated a cumulative distribution function (CDF) plot (Fig. 4*G*). The CDF characterizes the probability that a residue assumes a value less than or equal to a specified distance from the origin. A leftward shift of the CDF curve indicates a distribution concentrated near lower distance values, reflecting a higher probability mass close to the origin. Conversely, a rightward shift signifies a distribution concentrated toward higher distance values, indicating a greater probability mass farther from the origin. As seen in Figure 4*G*, there is a clear separation between the two CDF curves. The CDF corresponding to subtype-specific mutants was entirely right-shifted relative to that of the common mutants, indicating consistently greater distances from the origin. This pattern suggests that subtype-specific mutations are associated with more pronounced differences in the evaluated features, while common mutations exhibit minimal variation across subtypes. The lack of overlap between the CDFs underscores a strong separation between the two distributions, therefore supporting the hypothesis. When comparing the predictive power of individual properties, we observed that contact frequencies gave the clearest separation (Fig. S4*D*), followed by weighted degree (Fig. S4*E*) and then by interaction energies (Fig. S4*F*). Taken together, this supported our hypothesis and demonstrated the predictive power of the three computational features.

### Allosteric communication between functionally important regions in the 2 Gα subtypes

The activation of the Gα subunit involves coordinated motions across structural regions that are spatially distant, and prior studies have shown the presence of allosteric communication within the Gα subunit (13, 30). However, the mechanistic basis for allosteric communication between different structural regions of the Gα subunits and the residues involved in such communication are not known. Having identified conserved interface residue positions that exhibit subtype-specific impact on Gα:Gβγ dissociation, we next analyzed the mechanism of allosteric communication within the Gα protein using the BNM.

The edges in the BNM show codependency in dynamic movement between both spatially proximal residues and between allosteric or spatially distant residues. We calculated allosteric codependencies (edge strength) from BNM to identify distant residue pairs that show long range correlated movement. We summed up the edge strengths of all edges that connect residues from two structural regions to uncover how different structural regions show correlated movements. This would lead to an understanding of the allosteric communication mechanism within the Gα_i1_ and Gα_q_ subtypes. We constructed radial graphs (also known as circos maps) shown in Fig. S6 that show the total edge strengths between structural regions connecting local and distant residues in Gα_i1_ and Gα_q_. For both Gα subtypes studied here, we observed strong correlated motion or co-dependencies in the BNM between residues in the structurally neighboring regions, indicating impact of movement of neighboring residues in a local environment. Structural regions containing Gα:Gβγ interface residues (αD-αE loop, α2 helix, β4-α3 loop) showed correlated movement with distant (allosteric) residues within the interface. Furthermore, they also showed correlated movement to structural regions beyond the Gα:Gβγ interface, namely α1-β1 loop, α1-αA loop, αA helix, and β5-αG loop, all of which are distant from the Gα:Gβγ interface. Interestingly, while α5 of Gα_i1_ showed allosteric correlated movement only with other Ras-like domain regions (α2, α3, and α4-β6 loop), α5 of Gα_q_ also had allosteric correlated movement with AHD regions (α2, αC, αF). This is potentially significant given that the α5 helix extends into the receptor cavity and represents a major determinant of Gα coupling specificity. We also observed correlated movement between nucleotide binding site regions and the Gα-Gβγ interface (*i.e.*, between β1-α1 loop and β4-α3 loop), as affirmed by our previous study (13).

**Figure 5:**
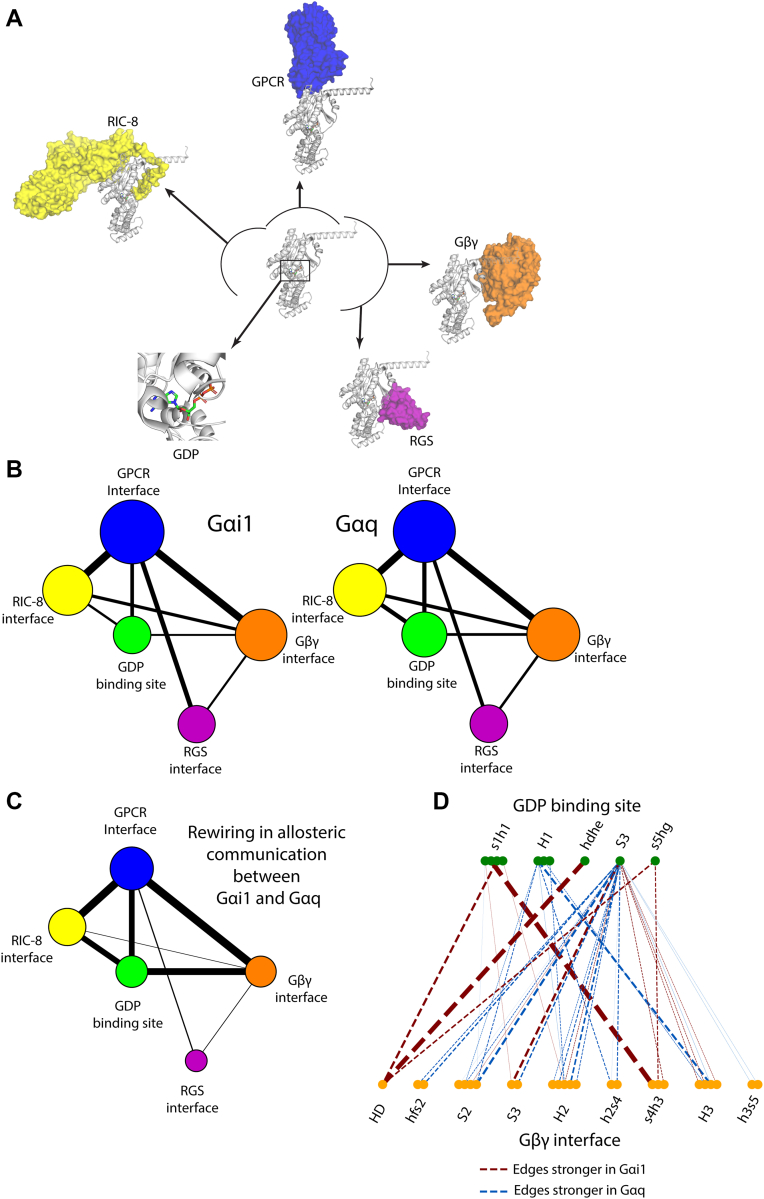
**BNMs show the Allosteric Communication between different protein interaction interface sites in Gα subunits.***A*, interface locations of proteins that are known to interact with the Gα subunit. In a clockwise direction from top: G protein-coupled receptor (GPCR), Adenylyl Cyclase, Gβγ dimer, Regulator of G protein Signaling (RGS), GDP, and guanine nucleotide exchange factor (RIC-8). *B*, meta network illustrating the correlated movements between the interfaces to the major interacting partners. *Blue*: GPCR interface, *Orange*: Gβγ interface, *Purple*: RGS interface, *White*: GDP binding site, *Yellow*: RIC-8 interface. The sizes of the meta nodes represent their weighted degrees, while the width of the meta edges represent their weights. *C*, rewiring of allosteric communication between Gα_i1_ and Gα_q_. Rewiring is defined as the summation of weighted hamming distances of all edges connecting the two regions, between Gα_i1_ and Gα_q_. The width of an edge represents the amount of rewiring happened across two regions between Gα_i1_ and Gα_q_. *D*, difference in the edge strengths between the same edges in the BNM networks for Gα_i1_ and Gα_q_ calculated as the Hamming distance. Hamming distance changes indicates the rearrangements of co-dependencies between GDP binding site and Gβγ interface (right). *Maroon*: edges that are stronger in Gα_i1_; *marine*: edges that are stronger in Gα_q_.

Having considered allosteric communication with receptors and nucleotides, we examined the existence of allosteric communication or correlated movement in regions where the Gα subunits bind to effectors and regulators. We constructed a multi-scale meta-network representation of BNM in Gα. We first delineated the Gα residues located in the interaction interfaces of Gα with well-characterized binding partner proteins, including GPCRs, Gβγ, Regulator of G-protein Signaling (RGS) proteins, and the guanine-nucleotide exchange factor or chaperone RIC-8, as well as with GDP (see Experimental procedures, Fig. 5*A*). The residues of Gα involved in these interfaces were calculated using GetContacts from the respective crystal structures (See Experimental procedures, Tables S2 and S3). Each “meta-node” corresponds to one of these interfaces (Fig. 5*B*), and each edge in the meta-network encodes the correlated motions calculated between pairs of interface residues (sum of all edge strengths). The edge weights in the meta-network were computed as the sum of all edge weights connecting residues belonging to the corresponding interface residue pairs. Weighted degrees of meta-nodes were obtained by summing the edge strengths of all meta-edges incident on that node. This abstraction enabled a compact yet informative summary of dependencies that link functionally active regions within each Gα subunit.

The resulting meta-network, or higher order structural level network, models for Gα_i1_ and Gα_q_ shown in Figure 5*B* displayed strikingly similar global topologies. In both subtypes, we observed strongly correlated motions between the residues in the GPCR:Gα interface and those in the Gα:Gβγ interface, followed by the GPCR:Gα interface to the RIC-8 interface and to the RGS interface. Whereas GPCRs and RIC-8 can function as guanine nucleotide exchange factors (GEFs) for Gα subunits (31), RGS proteins act as GTPase-activating proteins (GAPs) (32), collectively regulating the GTPase on-off cycle. The observed correlated movements among the GPCR, RIC-8, and RGS interfaces suggest that these regulatory sites are intrinsically coupled within the conformational landscape of the Gα subunit. Such coordinated dynamics likely reflect allosteric networks that facilitate transitions through nucleotide exchange and hydrolysis, even in the absence of bound effectors.

Although the total edge strength connecting residues across structural regions appears similar in Gα_q_ and Gα_i1_ (Fig. 5*B*), we observed substantial rewiring or reweighting of the allosteric dependencies at the level of individual residue pairs between different structural regions. To quantify these differences, we computed the weighted Hamming distance, which measures changes in corresponding edge strengths between the Bayesian network models of Gα_i1_ and Gα_q_ (Fig. 5*C*). The analysis revealed a significant difference (24.0 bits, *i.e.*, the number of differing edges), indicating that while the overall magnitude of allosteric communication to the Gβγ interface in the two subtypes is comparable, the underlying pathways are reorganized. To illustrate this, we examined edge weights linking the GDP-binding site to the Gβγ interface (Fig. 5*D*). We observed a rewiring of allosteric dependencies between these two structural regions. While most connections are stronger in Gα_q_, a subset of edges is stronger in Gα_i1_, reflecting distinct communication routes. Notably, the more distributed connectivity in Gα_q_ may underlie its heightened sensitivity to mutations: 14 of the 16 tested mutations significantly affected Gα_q_ dissociation.

## Discussion

Although conserved amino acids at the interface of proteins are assumed to have similar effect, our study revealed that certain conserved amino acids on Gα_q_ and Gα_i1_ subtypes show differential effects on coupling to the Gβγ subunits. These differences primarily stem from the dynamics of the Gα subunit. By integrating three complementary features derived from MD simulations, namely: (i) residue contact frequencies, (ii) residue interaction energies, and (iii) co-dependencies among the residue pairs derived from Bayesian Network Analysis, we showed that the subtype selective coupling of Gα_i1_ and Gα_q_ comes from a complex confluence of temporal persistence of residue contacts, as well as from enthalpic and cooperative effects. This triangulation revealed subtype-specific interface characteristics that became apparent only from the dynamic ensemble analysis. Through an iteration of prediction and experimental testing we have shown that conserved residue positions can elicit distinct functional outcomes in paralogous proteins.

Switch II (α2 helix) and switch III (α3 helix) make direct contacts with Gβγ and undergo substantial conformational changes upon Gα activation. Therefore, residues within these two regions have been the central focus of prior studies investigating determinants of Gβγ release. Consistent with previous studies (12, 13), mutations at Switch II and III were disruptive in both Gα subtypes in our study. Beyond the two canonical regions, our approach also highlighted previously understudied structural regions of the AHD and identified hotspot residues that affect Gβγ coupling upon mutation in Gα_q_ but not in Gα_i1_. These observations suggest that the AHD, a region primarily known for its interaction with RGS and its function in facilitating nucleotide exchange and hydrolysis (33, 34, 35), may play a pivotal role in Gβγ dissociation. Indeed, subtype-dependent differences we discovered in this region are consistent with previously reported differences observed in BRET-based measures of Gβγ release and rebinding (36). Those hotspots in the AHD, along with a few others in the Ras-like domain, are clustered around the Ras-AHD clamp region. This clamp region mediates the opening and closing motion of the Ras domain and AHD that is necessary for nucleotide exchange during Gα activation. We speculate that these differences in the clamp regions can also lead to differences in nucleotide exchange rates (28, 37, 38). The recovery of previously reported hotspots, together with the discovery of new subtype-specific hotspots, provides internal support for our computational pipeline, which combines classical physical interaction metrics with a probabilistic machine learning network model to estimate cooperativity in coupling (12, 13).

Two caveats are worth mentioning. First, the BRET assay employed Gβγ subtypes, which differ from those used to start MD simulations: Gβ_1_ and Gγ_2_ have been the model subunits for heterotrimeric G protein crystallization, while Gβ_3_ and Gγ_9_ have been shown to provide a robust BRET signal (27). The other caveat is that while we focused on conserved interface residues in Gα, the contacting residues in Gβ and Gγ are not all the same. This should be considered when interpreting the BRET readout.

Our computational features capture both the effect of local environments and allosteric communication of the interface residues. A previous study by Hewitt-Valentin *et al.* has shown that the conserved “catalytic Gln” residue that is outside the Gα:Gβγ interface showed subtype-specific effects on Gα_q_ and Gα_i1_, and these effects were the result of long-range allostery (1). The present study focuses on conserved residues within the interface because they provide the most rigorous test of whether spatially equivalent conserved residues produce nonequivalent outcomes across paralogs. Building on our new and previous work (1), future studies will examine the role of conserved residues outside the Gα:Gβγ interface and their allosteric contributions to differential Gα:Gβγ coupling, thereby extending the scope beyond the central questions addressed here.

Finally, our results highlight the need to consider G protein mutations within the broader network of receptor–effector interactions, rather than as isolated perturbations of a given signaling component. Furthermore, twelve of the subtype-specific hotspots have reported disease-associated mutations in either subtype, signifying the functional importance of those positions (39, 40, 41). In this context, it is important to consider whether a disease-associated mutation in one G protein subtype will necessarily have the same effect in another subtype. For example, mutations may disrupt coupling to some cognate binding partners but not others, or enable new interactions with non-cognate proteins, effectively reshaping the downstream signaling network. More broadly, these findings indicate that paralogous signaling proteins may harbor subtype-specific mutational sensitivities even when their sequences and global structures are largely conserved. Our strategy of integrating interface energetics, cooperative coupling, and mutational assessment is broadly applicable to other paralogous proteins. As efforts toward precision pharmacology increasingly consider the role of protein-protein coupling selectivity, analytical approaches capable of identifying non-obvious determinants of specificity may prove valuable for future therapeutic design.

## Experimental procedures

### Multiple sequence alignment

The sequences of human Gα_i1_ and Gα_q_ were retrieved from UniProt (https://www.uniprot.org/) with accession code P63096 and P50148, respectively. The alignment was performed using ClustalW web service (https://www.genome.jp/tools-bin/clustalw) and visualized using Jalview (42). For the alignment of the interface residues, the interface residues were extracted from the contact results and aligned using the same procedure as the full sequence alignment.

### Details of molecular dynamics (MD) simulations

The structure of GDP-bound Gα_i1_-Gβγ complex was downloaded from RCSB (PDB ID: 6CRK). To generate a structural model for the GDP-bound inactive state of Gα_q_:Gβγ complex, we downloaded the structure of apo Gα_q_-Gβγ complex from RCSB (PDB ID: 7F6G), in which the AHD of Gα_q_ was focus resolved. GDP was transferred to this Gα_q_ structure from 6CRK, by aligning Gα_i1_ and Gα_q_ by their Ras-like domain. The αN helix was removed from both structures; Because the simulations were performed in solution, without a membrane present, the αN helix, which requires membrane anchoring for stability, was removed from both structures. We optimized the side chain rotamer conformations of the residues within 7 Å of GDP, followed by energy minimization using the conjugate gradient method with a convergence cutoff of 0.1 kcal/mol Å^2^. Both operations were performed using the Prime module of Maestro software from Schrodinger (https://www.schrodinger.com/products/maestro). Input structure files for MD simulations were generated using CHARMM-GUI (43). Each complex was solvated in explicit transferable intermolecular potential with 3 points (TIP3P) water molecules in a cubic box (11.5 nm × 11.5 nm × 11.5 nm) with 0.15 M of potassium chloride for maintaining the physiological condition. We used software GROMACS (44) (Version 2022.3) with all-atom CHARMM36m force field (45) to perform MD simulations at 310 K coupled to a temperature bath with a relaxation time of 0.1 ps (46). Desired pressure for all systems were achieved by using Parrinello-Rahman barostat with a pressure relaxation time of 2 ps (47) Equilibrium bond length and geometry of water molecules were constrained using the SHAKE algorithm (48). The short-range electrostatic and van der Waals interactions were estimated every 2 fs timestep using a charged group pair list with a cutoff of 8 Å between centers of geometry of charged groups. Long-range van der Waals interactions were calculated using a cutoff of 14 Å and long-range electrostatic interactions were treated with the particle mesh Ewald method (49). Temperature was kept constant at 310 K by applying the Nose-Hoover thermostat (50). Before production runs, we equilibrated the systems using the following procedure: All systems were subjected to a 5000-step steepest descent energy minimization to remove clashes (51). After minimization, the systems were heated up to 310 K under a constant number-volume-temperature ensemble (NVT). The simulations were saved every 200 ps for analysis. The protein and nucleotide were subjected to positional constraints under a harmonic force constant of 1000 kJ/(mol∗nm^2^) during the NVT step, while solvent molecules were free to move. The systems were then further equilibrated using constant pressure ensemble (NPT), in which the force constant applied to the protein, Mg ion, and nucleotide were gradually reduced from 5 kJ/(mol∗nm^2^) to zero in six steps of 5 ns each. An additional 50 ns of unconstrained simulation was performed, making it a total of 80 ns NPT equilibration prior to production runs. We performed five production runs of 1000 ns each using five different initial velocities for every system. We collected the five trajectories to obtain a 5 μs MD trajectory for both Gα_i1_-Gβγ complex and Gα_q_-Gβγ complex. The convergence of each MD trajectory was validated by plotting the time series of the RMSD in the coordinates of the backbone atoms of the G protein trimer with respect to the starting structure of production run (Fig. S7). All the runs show a RMSD converging at around 7 Å and most of these RMSD come from the relative movement of the Ras-like domain and AHD and movements of Gβγ N termini.

### Per-residue contact frequency of Gα residues

For each system, the last 600 ns from five independent MD simulation runs were merged into one concatenated trajectory. The concatenated trajectory was sampled every 2 ns. The sampled trajectory was fed to GetContacts script available on GitHub (https://github.com/getcontacts/getcontacts). For specific parameters, the interaction type flag “itype” was set to “all”, and the two atom group flags for contacts were “chain A” and “chain B or chain G”. Contact results were further processed in Python environment: briefly, the contact frequency of a Gα residue is calculated as the percentage of MD frames in which said residue makes any contact with any residue on Gβ protein or Gγ protein. The contact frequencies were used to generate Fig. 2*C*. Residues with contact frequencies greater than 20% were used for plotting Fig. S2*B*.

### Per-residue interaction energy of Gα residues

For each system, we aggregated the last 600 ns of each trajectory run to calculate the pair-wise interaction energies of contact pairs between Gα and Gβγ, sampled every 2 ns. For analyzing the interface residues, each residue of a contact pair was grouped into an energy group in GROMACS, and the total non-bond interaction energy (van der Waals + Columbic) was calculated using gmx energy module. The per-residue interaction energy of a residue was calculated by summing the interaction energies of all contact pairs in which said residue was involved.

### Bayesian Network Model generation

The BNM was constructed using BaNDyT (19) software developed by us, using the workflow shown in Figure 3*A*. The input feature to generate the BNM was the interaction energy of each residue with all the residues within 12 Å in each MD snapshot. For each G protein system, we aggregated the last 600 ns of each of the five trajectories to calculate the pair-wise interaction energies of contact pairs between Gα and Gβγ, sampled every 2 ns and calculated the per-residue interaction energy for each MD snapshot. The interaction energies were assembled in a matrix with each row representing a frame from the trajectory, and each column representing a residue (Fig. 3*A*). The two matrices acquired from MD trajectories of Gα_i1_ and Gα_q_ were stacked vertically, keeping only the shared columns. The distribution of the interaction energies for each residue across the MD simulation trajectories given in the concatenated matrix was discretized using MaxEnt into eight bins. This was fed to the BANDyT workflow to generate a Bayesian network universal graph (52). Once the universal graph was recovered, the individual networks were obtained by rescoring against stratified data, using mutual information between the nodes. The output was further processed in Python environment.

The network was visualized using Gephi (https://github.com/gephi). The network was saved in graphml format and fed to Gephi. The weighted degree for each node (residue in Gα subunit) was calculated as the sum of all the edge strengths connected to the given node (see Fig. 3*A*). The color and the size of the nodes are proportional to the weighted degree; the width and color of the edges were proportional to the edge weight.

For meta-network generation, the residue-level network was coarse-grained by summing residues into groups (Gα:Gβγ interface, GDP-binding site, GPCR interface, and RIC-8 interface). The interfaces were defined as follows: Gα:Gβγ interface was defined by GetContacts, described above. GDP-binding site was defined by GetContacts; specifically, the topology and trajectory used were the same as described in section “*Per-residue contact frequency of Gα residues*”, the group selection used were “resname GDP” and “chain A”, with “itypes” flag set to “all”. The definition of GPCR interface, adenylyl cyclase interface, RGS interface, and RIC-8 interface was taken from a previous publication (53). Briefly, the protein entity information was retrieved from RCSB (www.rcsb.org), and GetContacts was used to calculate the interacting residues between the Gα subunit and its partner proteins. The interface residues can be found in Table S2. The PDB structures used can be found in Table S3. Each structural region forms a meta-node, and the meta-edges represent the summed pairwise edge weights between residues in the corresponding regions. Meta-node size is proportional to residue count, and meta-edge width corresponds to the aggregated inter-region connectivity. Ras-other and AHD-other encompass residues that are in Ras and AHD, respectively, but are not part of the functionally defining regions.

Weighted hamming distances were calculated using the Python NetworkX package. The weighted hamming distances of all edges across two regions were summed and used as the amount of signal rewiring across two regions.

### Robustness test for Bayesian Network Model

The concatenated trajectory comprised 10,000 frames organized into five independent segments of 2000 frames, each corresponding to an independently initialized velocity. Bootstrap resampling was performed with replacement within each segment, preserving the original segmentation. One thousand bootstrap ensembles were generated. For each ensemble, network edges were re-estimated and scored against the universal graph using mutual information between node pairs. The confidence interval was calculated as an evaluation. The relevant data can be found in the Supporting information matrix 1 (weighted degree edge confidence interval) and Supporting information matrix 2 (weighted hamming distance edge confidence interval).

### Bioluminescence resonance energy transfer (TRUPATH BRET)

HEK293T/17 cells (ATCC; CRL-11268) were maintained at 37 °C, 5% CO_2_ in a HERAcell VIOS 160i CO_2_ incubator (Thermo Fisher Scientific; 51033547) in Dulbecco’s Modified Eagle Medium (DMEM (Corning; 10013CV) with 10% fetal bovine serum (Sigma-Aldrich; F2442), 100 U/ml penicillin, and 100 μg/ml streptomycin (Gibco; 15140122). 5 to 8 h after seeding (1,000,000 cells per 3 ml), the cells were transfected with 300 ng human neurotensin receptor 1 (NTS1R), Renilla luciferase 8 (Rluc8) tagged human Gɑ_i1_ or Gɑ_q_, human Gβ_3_, and green fluorescent protein 2 (GFP2) tagged human Gγ_9_ in a 1:1:1:1 ratio using TransIT-2020 transfection reagent (MirusBio; MIR 5404) (1). 14-18h post-transfection, the cells were seeded on a 96-well plate (Corning; ref 3903) coated with poly-D-lysine (Gibco; A38904-01) at a density of 80,000 cells in 150 μl 1% FBS and 1% PEN/STREP DMEM per well. The next day, the plates were washed with 60 μl per well of drug buffer (20 mM 4-(2-hydroxyethyl)-1-piperazineethanesulfonic acid (HEPES) (Thermo Fisher Scientific; J16924.K2), 0.3% bovine serum albumin (Sigma-Aldrich; A6003), 0.03% L-ascorbic acid (Sigma-Aldrich; A92902), in Hanks’ Balanced Salt Solution (Gibco; 14025092), pH 7.4) for 10 min at 37˚C before the addition of Rluc8 substrate, coelentrazine 400a (NanoLight; 340), at 7.5 μM (1.5×) in 60 μl drug buffer. After a 10 min incubation in the dark at room temperature, 30 μl (3×) of neurotensin (Tocris; 1909) in drug buffer was added to the plate for final well concentrations ranging from 100 μM to 0 μM. Following a 10 min dark incubation, the plate luminescence was read by a CLARIOstar Plus (BMG LABTECH) in a 4 mm spiral well scan at 410 nm (Rluc8 bioluminescence) and 530 nm (GFP2 fluorescence). The BRET (GFP2 emission/Rluc8 emission) ratio was baseline corrected in GraphPad Prism 10 (GraphPad Software Inc.) by subtracting the respective zero agonist BRET ratio from each sample to yield net BRET. Each mutant is then normalized to its respective wildtype (Gɑ_i1_ or Gɑ_q_)—with the wild-type basal BRET ratio being 0% response and the minimum wild-type BRET ratio (maximum agonist-induced response) set to 100% response.

The basal BRET values of mutants were normalized to their respective wild-type by setting the wild-type value to 1 and rescaling all mutants accordingly, and plotted in Fig. S8.

The dose-response curves in Figure 4*D* and Fig. S5 were plotted in GraphPad Prism v10.6.1. The curves were generated by a non-linear fit using lo (agonist) vs. response mode. Coloring and labelling of the curves were done in Adobe Illustrator.

### Figure generation of computational data

Heatmap was generated using Seaborn package in Python. We used the color map “plasma”, with the lower bound set to 20% for contact, and the maximum value for interaction energy and the upper bound set to 100% for contact, and the minimum value for interaction energy. The labels of heatmaps were generated in Adobe Illustrator.

PYMOL figures were generated using the plotting array of the heatmap. The Cα atoms of the residues were colored according to their values. We adopted the same color map as the heatmap. The nucleotide was colored per atom type, with carbon set to white (pymol.cmd.util.cbaw).

The circos maps of the BNM were generated using Python Holoview package. The nodes were ordered according to the sequence and labeled using their region names. The labels were further processed in Adobe Illustrator.

### Statistical analysis

For contact frequency and interaction energy, the values from each frame were compiled as an array, and a two-tailed Welch’s *t* test was applied to calculate the *p*-value. *p*-values less than 0.05 were considered statistically significant.

## Data availability

The MD trajectories are available on GPCRmd.org. They will be made available upon acceptance.

## Supporting information

This article contains supporting information.

## Conflict of interest

The authors declare that they have no conflicts of interest with the contents of this article.

## Supporting information

Supporting Figures S1-S8 and Tables S1–S3

Supporting information matrix 1

Supporting information matrix 2
